# Implications of institutional racism in the therapeutic itinerary of people with chronic renal failure

**DOI:** 10.17533/udea.iee.v38n2e09

**Published:** 2020-07-10

**Authors:** Ricardo Bruno Santos Ferreira, Climene Laura de Camargo, Maria Inês da Silva Barbosa, Maria Lúcia Silva Servo, Marcia Maria Carneiro Oliveira, Juliana Alves Leite Leal

**Affiliations:** 1 Nurse, Masters. Professor at Universidade do Estado da Bahia, Brazil and Nurse at the Kidney Hospital of Guanambi, Bahia, Brazil. Email: ricardobrunoenf@gmail.com. Corresponding author Universidade do Estado da Bahia Universidade do Estado da Bahia Brazil ricardobrunoenf@gmail.com; 2 Nurse, Ph.D. Full Professor at Universidade Federal da Bahia, Brazil. Email: climenecamargo@hotmail.com. Universidade Federal da Bahia Universidade Federal da Bahia Brazil climenecamargo@hotmail.com.; 3 Social Worker, PhD. Retired Professor of Universidade Federal do Mato Grosso, Brazil. Email: maria.br@terra.com.br Universidade Federal do Mato Grosso Universidade Federal do Mato Grosso Brazil maria.br@terra.com.br; 4 Nurse, Ph.D. Full Professor at Universidade Estadual de Feira de Santana, Brazil. Email: mlsservo@uefs.br Universidade Estadual de Feira de Santana Universidade Estadual de Feira de Santana Brazil mlsservo@uefs.br; 5 Nurse, Ph.D. Assistant Professor at Universidade Federal da Bahia, Brazil. Email: marcia.carneiro@ufba.br Universidade Federal da Bahia Universidade Federal da Bahia Brazil marcia.carneiro@ufba.br; 6 Nurse, Ph.D. Adjunct Professor at Universidade Estadual de Feira de Santana, Brazil. Email: julileite@hotmail.com Universidade Estadual de Feira de Santana Universidade Estadual de Feira de Santana Brazil julileite@hotmail.com

**Keywords:** racismo, renal insufficiency, chronic, health services accessibility, ethnic inequality., racismo, insuficiencia renal crónica, accesibilidad a los servicios de salud, inequidad étnica., racismo, insuficiência renal crónica, acesso aos serviços de saúde, iniquidade étnica.

## Abstract

**Objective.:**

To understand the implications of institutional racism in the therapeutic itinerary of patients with chronic renal failure (CRF) in the search for diagnosis and treatment of the disease.

**Methods.:**

Descriptive, qualitative study developed with 23 people with CRF in a regional reference hospital for hemodialysis treatment in Northeast Brazil. Two techniques of data collection were used: semi-structured interview and consultation to the NEFRODATA electronic medical record. For systematization and analysis, the technique of content analysis was used.

**Results.:**

Black and white people with CRF showed significant divergences and differences in their therapeutic itineraries: while white people had access to diagnosis during outpatient care in other medical specialties, black people were only diagnosed during hospitalization. In addition, white people had more access to private health plans when compared to black people, which doubles the possibility of access to health services. Moreover, even when the characteristics in the itinerary of black and white people were convergent, access to diagnosis and treatment proved to be more difficult for black people.

**Conclusion.:**

The study showed the presence of institutional racism in the therapeutic itinerary of people with kidney disease in which black people have greater difficulty in accessing health services. In this sense, there is a need to create strategies to face institutional racism and to consolidate the National Policy for Comprehensive Health Care of the Black Population.

## Resumen

**Objetivo.:**

Comprender las implicaciones del racismo institucional en el itinerario terapéutico de pacientes con insuficiencia renal crónica (IRC) en la búsqueda del diagnóstico y tratamiento de la enfermedad.

**Métodos.:**

Estudio descriptivo, cualitativo, desarrollado con la participación de 23 personas con IRC en un hospital regional de referencia para tratamiento de hemodiálisis en el noreste de Brasil. Se utilizaron dos técnicas de recolección de datos: entrevista semiestructurada y consulta a la historia clínica electrónica NEFRODATA. Para la sistematización y el análisis, se utilizó la técnica de análisis de contenido.

**Resultados.:**

Las personas negras y blancas con IRC mostraron divergencias y diferencias en sus itinerarios terapéuticos: mientras que las personas blancas tuvieron acceso al diagnóstico durante la atención ambulatoria con otra especialidad médica, las personas negras solo fueron diagnosticadas durante la hospitalización. Además, las personas blancas tenían más acceso a planes de salud privados en comparación con las personas negras, lo que les brindaba una doble posibilidad de acceso a los servicios de salud. También se agrega que, incluso, cuando las características en el itinerario de las personas blancas y negras eran convergentes, el acceso al diagnóstico y al tratamiento fue más difícil para las personas negras.

**Conclusión.:**

El estudio evidenció la presencia de racismo institucional en el itinerario terapéutico de las personas con enfermedad renal, en el que las personas negras tienen mayor dificultad para acceder a los servicios de salud. En este sentido, es necesario crear estrategias para enfrentar el racismo institucional y consolidar la Política Nacional para la Salud Integral de la Población Negra.

## Resumo

**Objetivo.:**

Compreender as implicações do racismo institucional no itinerário terapêutico de pacientes com insuficiência renal crônica (IRC) na busca pelo diagnóstico e tratamento da doença.

**Métodos.:**

Estudo descritivo, qualitativo, desenvolvido com 23 pessoas com IRC em um hospital de referência regional para tratamento hemodialítico no Nordeste do Brasil. Foi utilizado duas técnicas de coleta de dados: entrevista semiestruturada e consulta ao prontuário eletrônico NEFRODATA. Para sistematização e análise utilizou-se a técnica de análise de conteúdo.

**Resultados.:**

As pessoas negras e brancas com IRC apresentaram divergências e diferenças em seus itinerários terapêuticos: enquanto as pessoas brancas tiveram acesso ao diagnóstico durante o atendimento ambulatorial com outra especialidade médica, as pessoas negras só foram diagnosticadas durante internação hospitalar. Ademais, as pessoas brancas tiveram mais acesso a planos privados de saúde quando comparados às pessoas negras, o que confere dupla possibilidade de acesso aos serviços de saúde. Acrescenta-se ainda que, mesmo quando as características no itinerário das pessoas negras e brancas foram convergentes, o acesso ao diagnóstico e tratamento se mostrou mais dificultado para pessoas negras.

**Conclusão.:**

O estudo evidenciou a presença de racismo institucional no itinerário terapêutico de pessoas com doença renal, em que pessoas negras possuem maior dificuldade de acesso aos serviços de saúde. Nesse sentido, há necessidade de criar estratégias para o enfrentamento do racismo institucional e para consolidação da Política Nacional de Saúde Integral da População Negra.

## Introduction

The demographic transition process evidenced in Brazilian society in recent years has presented challenges for federal management, especially with regard to new health needs as a result of the change in the morbidity and mortality profile. In this context, there has been a reduction in deaths from infectious diseases and a progressive increase in chronic non-communicable diseases.(1) Chronic Renal Failure (CRF) is part of the list of chronic non-communicable diseases that had a significant increase in incidence and prevalence. This condition is understood as the final stage of chronic kidney disease, when the kidney is unable to maintain hydroelectrolytic balance and patients begin to require kidney replacement therapy. It is a progressive disease whose main risk factors are systemic arterial hypertension, diabetes mellitus and family history.(2) Treatment can be carried out using three therapeutic modalities: peritoneal dialysis, hemodialysis and kidney transplantation.(3) However, despite treatment, the mortality of these people is high, being higher than 20% in the first year of dialysis.(4) It is believed that this high percentage of deaths at the onset of treatment is related to difficulties to access early diagnosis.(3)

The increase in incidence was confirmed in the Brazilian Chronic Dialysis Survey, which detected an increase of 31 500 patients on dialysis from 2012 to 2016, reaching a total of 122, 825 people with CRF at the end of 2016.(5) Stresses it is said that this increase in the onset of the disease is evidenced mainly in the black population, in which the incidence rate is three times higher when compared to white people.(6) International studies also pointed out that the prevalence of CRF is four times higher in people of African ethnicity when compared to those of European ethnicity.(7) Something similar was found in the United States, where kidney disease predominantly affects African Americans.(8) The problem extends to kidney transplantation, a type of kidney replacement therapy that increases the quality of life of patients. When compared to white patients, African-Americans have less than half the chance of having access to this therapeutic modality.(4)

It is noteworthy that the literature often shows studies that discuss the prevalence of CRF and its main underlying diseases, such as diabetic nephropathy, only from a biological perspective, justifying the higher prevalence among black people with genetic predisposition,(9) but overlooking the worsening of the condition that results from the precarious living conditions of this population. Furthermore, it is essential to highlight that CRF has a more serious evolution or difficult treatment in the black population as a result of the structuring racism that impacts on the health conditions of this population contingent.(10) It is added that racism not only impacts the birth, life and death conditions of black people, but also produces and reproduces unequal access due to race/skin color.(11) When racism is institutionalized, it acts as a determinant for the onset of the disease, search for diagnosis and treatment of CRF.

As it is a progressive and asymptomatic disease in the early stages, broad access to health services must be present throughout the paths taken by patients so that early diagnosis and correct treatment be possible. The path taken by patients in the search for the diagnosis and treatment of certain pathologies is called the therapeutic itinerary (TI).(12) It is believed that the study of TIs of black and white people with CRF can be used to help the health teams that follow-up this population to understand the racial, social and cultural context in which these individuals are inserted. This type of study also contributes to identify factors that facilitate and/or hinder the access to the health services of each group, aiming to contribute to decision making regarding the approaches adopted in different lines of care. In this sense, this study aims to understand the implications of institutional racism in the therapeutic itinerary of patients with CRF in the search for diagnosis and treatment of the disease.

## Methods

The study is a product of the master’s thesis of the first author of the study entitled “Implications of racism in the access of people with chronic kidney disease to health services”, developed in 2019 in the Department of Masters in Professional Nursing (MPE) of the Health Department at the State University of Feira de Santana (UEFS), Bahia, Brazil. As a characteristic of the MPE, questions about the theme emerged after observations in the reality experienced while assisting people with CRF, most of whom were of black race/skin color.

This is a descriptive study with a qualitative approach developed in a regional reference hospital for hemodialysis treatment, located in a municipality in the northeast of Brazil. This hospital unit is a private law institution affiliated to the Unified Health System (SUS) and private health plans. The service serves people diagnosed with acute and CRF, and provide conservative follow-up for CRF patients in the non-dialysis phase.

The study included 23 people diagnosed with CRF over the age of 18 who undergo hemodialysis at the institution. People diagnosed with acute CRF and people with chronic CRF in the non-dialysis phase were excluded. Data collection took place in the months of June and July 2019. Patients were randomly approached in the waiting room before the treatment was performed, and the study was presented and, after their signing of the informed consent form, the interview was conducted. Two techniques were used for data collection: interview and consultation of secondary data through electronic medical records.

Two data collection instruments were used: a semi-structured interview accompanied by a script containing closed questions about sociodemographic aspects and open questions about the object studied, and a script for the collection of clinical information in medical records. Regarding the sociodemographic characterization of the participants, the information sought was: sex, race/skin color, education, income, receipt of social benefits, way of admission to the nephrology service, and access to conservative treatment. For the interviews, the guiding questions were: tell me what happened to you until you found out you had CRF; describe the facilitators and barriers faced to access diagnosis and treatment. The interviews were recorded and transcribed verbatim for analysis of their content.

Data collection was completed when empirical information saturation was reached. After the transcription, the interviews were returned to the participants for comments and/or corrections. In order to guarantee rigor in qualitative research, the checklist of qualitative research was adopted in all stages of the 32 items present in the guidelines of the Consolidated Criteria for Reporting Qualitative Research (COREQ). Data were organized in two tables based on the self-declared race/skin color: blacks and whites. Then, a comparative chart of the itinerary of the groups was elaborated, organized from the convergences, divergences and complementarities, so that it was possible to understand the manifestations of racism and its implications in the patients’ therapeutic itinerary. This systematization followed the characteristics of the proposed content analysis, being treated from the stages of pre-analysis with transcription and quick reading of the interviews; exploration of the material with codification and structuring of convergences, divergences and complementarities; and treatment of results with construction of inferences and confrontation with data in the existing literature.

The study complied with ethical precepts with respect to the interviewees’ autonomy, confidentiality of data, and acceptance to participate in the study by reading and signing the informed consent form. To guarantee anonymity, the participants were identified by the letter “I”, followed by a number that corresponded to the order of the interview and respective self-declared race/skin color. The study was approved by the UEFS Research and Ethics Committee with CAAE 90202618.0.0000.0053.

## Results

Among the 23 study participants, 52.2% were women, 52.8% declared to be white and 48.2% black. Regarding education, 25% of white patients attended higher education, while only 9.1% of black patients had had access to superior education. With regard to income, 81.8% of black people and 66% of white people had an income of up to 1 minimum wage (US $ 235 = R $ 1045 reais). In addition, only 18.2% of the black participants did not receive the benefit of continued provision for low-income people, while 41.7% of the white people did not receive this benefit.

Regarding the way of admission to the nephrology service, 81.8% of the black people were referred after the symptoms worsened and after hospitalization, while 58.3% of white people were referred from outpatient care (Family Health Strategy - FHS, conservative treatment or other medical specialties). It is also noteworthy that, among cases of outpatient care, only white people had access to follow-up at the FHS (8.3%) and conservative treatment (50%), which correspond to outpatient follow-up with a specialized nephrology team before the start of the hemodialysis.

The interviews revealed that people with different racial characteristics had different therapeutic itineraries. Table 1, described below, presents in a systematic way the aspects of the content of the speeches of black and white people, structured from the convergences, divergences and differences of the speeches.

**Table 1:** Systematization of the therapeutic itinerary of black and white people with CRF

Convergences	1 - Assistance resulting from the exacerbation of symptoms.
Convergences	2 - Chronic renal insufficiency as a secondary disease.
Convergences	3 - Lack of basic care in the therapeutic itinerary.
Convergences	4 - Presence of the private sector in the search for diagnosis.
Divergences	1 - Conservative treatment exclusively in the case of white people.
Divergences	2 - Access to private health plans exclusively in the case of white people.
Differences	1 - Access to diagnosis resulting from care in other specialties: white people during outpatient care and black people during in-hospital care.

It was noted that the therapeutic itinerary for both black and white patients began with the worsening of the disease and exacerbation of symptoms, manifested with malaise, shortness of breath and generalized edema: *I was started feeling bad at home, very short of breath and very swollen, then they took me to the hospital and there they found out I had the disease* (I3, black); *I started to swell, first it was the legs, then it was the face. That was how I found out. I did the test and found that I had a kidney problem*. (I20, white).

Another convergence between people of black and white race/skin color was the inexistence of basic care in the therapeutic itinerary of these people, and thus the diagnosis and referral resulted from in-hospital care. Difficulty in accessing the diagnosis was also evident, since the patients needed multiple consultations in different cities: (...) *My blood pressure was 18/14 and I stayed at the UPA all the afternoon. I left with the referral because I did several tests there and they found nothing. Then I went to Vitória da Conquista, because it was a more developed city. I had several consultations, I went to several doctors, but everything was private and they found nothing. My condition got worse and I couldn’t eat anymore, that was when I decided to go to Goiânia. I repeated all the exams until the nephrologist confirmed that I had kidney disease* (I7, black); *I already had a problem with my uterus, I was already being monitored by her [gynecologist] and I arrived a with very high blood pressure, then she said: you can’t be like that and sent me to the cardiologist. He was investigating, investigating, then on the ultrasound he requested (...) there was [kidney problem]* (I23, white).

The lack of basic care in the itinerary implied the presence of the private sector in the search for diagnosis of black and white people. In this sense, there is a difficulty in access that is directly associated with the enormous fragmentation of care, high financial cost for correct follow-up, and low resolution in small cities: *It was complicated because I spent a lot. Until I arrived in Goiânia I went to several doctors, did several tests and had to pay for everything. I paid R$ 300 reais for a consultation and the doctor said that he could not help, I even spent R$ 1,000 reais on exams and could not find a solution* (I7, black); *The whole treatment was private, until the moment I was admitted because I had very swollen feet* (I4, black)*; (...) I only use the FHS service during the vaccination campaign. I don’t do routine monitoring* (I14, white).

This high cost is directly related to fragmented assistance and a strong presence of the private sector. However, the private sector is only present in the itinerary until diagnosis. After the discovery of the disease, SUS becomes the main agent providing care, because the cost of treatment is high. Another converging characteristic between the two racial groups is that many patients had access to the diagnosis of CRF from the existence of another disease: *I did not feel the kidney first, I felt the heart first. I had a consultation with the Cuban doctor and he said: 'go to Carinhanha fast because your heart is swollen'. I was feeling short of breath, short of breath, anything I did caused me short of breath. Then I came to the hospital in Carinhanha, spent one night, and the next day early, they sent me to Guanambi. I was admitted to the hospital for 10 days and they found that because of my heart [problem] the kidney was affected* (I5, black); *my problem was caused by schistosomiasis, I was undergoing treatment for schistosomiasis, you know that it causes water belly, right? Then my belly started to grow and I went to São Paulo. When I got there, I had two surgeries, I removed the spleen, but the nephrologist said that I had a kidney problem, with beginning of nephritis* (I21, white).

On the other hand, a marked divergence between black and white people was that only the latter underwent conservative outpatient treatment before the start of kidney replacement therapy: *I did not do it [previous follow-up]. My follow-up started during the treatment. So much so that I went through several doctors and nobody found out, when I got sick and very bad that they took me to* Conquista *and I found out. When I realized I was already in the machine* (I1, black); *I did [follow-up] here with nephrologist since 2013 (...) I started to do a check-up every 3 months, it depended a lot on the tests he requested. When I made had the consultation, he would already give me the exams for me to do and the next time I would bring them [the results]* (I23, white).

In addition to the the fact that only white people did conservative treatment, another divergence between the two racial groups was that only white patients had access to supplementary health through private health plans, which facilitated access to follow-up, tests and treatment: *The kidney had stopped working with antibiotics, my kidney was stopped. I went back to the doctor who had evaluated me years before, I made a health plan that had a promotion in which there was immediate start of coverage, then I scheduled the consultation and went* (I19, white); (...) *and then I spent 4 years being followed-up in Montes Claros, but the consultation was private, I had the* planserv *that only provided care in Bahia. Then I did the private consultation and the exams she passed I did here in Bahia using the health plan. I never used SUS* (I16, white).

It was also perceived, especially in the statements of I16, the limits of private care, since health plans do not have universal coverage, which makes access spaces limited and predetermined.

## Discussion

Two analytical categories emerged from the analysis of the chart: 1) Fragmentation of care and racism involved in the convergences of the pilgrimage of people with renal failure; 2) Divergences and differences in the therapeutic itinerary of black and white patients and racism materialized in the lack of access for black people.

### Fragmentation of care and racism involved in the convergences of the pilgrimage of people with renal failure

The search for assistance goes through the representation that the groups have about the meaning of health.(13) Thus, the fact that black and non-black people seek assistance after the exacerbation of symptoms represents, in part, a limited possibility of taking care of the health, where the search for curative care occurs to the detriment of preventive care. We can affirm, given the statements, that the search for services aimed at health promotion and disease prevention is something that escapes the role of valuing this population, considering that the service health care is sought only in times of real need (urgent and emergency situations).

Furthermore, both the fact that the discovery of CRF was made during the treatment of a secondary disease, as well as the search for care when there was exacerbation of symptoms points to the lack of primary care as an integral part of the therapeutic itinerary of black and white patients. It should be noted that, according to ordinance 389 of March 13, 2014, the FHS has the responsibility to develop actions to prevent risk factors related to CRF, perform early diagnosis and refer patients to specialized care when diagnosis is confirmed. In addition, it is up to the FHS to define the criteria for the organization of care for people with CRF.(14) In this sense, we believe that the absence of the FHS in the itinerary has a more significant impact on the assistance to black people because this public presented a greater economic vulnerability and fewer years of schooling, found in the sociodemographic description, which implies less possibility of access to health services.

We believe that the lack of FHS in the patients’ itinerary negatively impacted the search for the consolidation of the National Policy for Comprehensive Health Care of the Black Population (PNSIPN). The PNSIPN focus the attention on diseases which are prevalent in the black population, especially diabetes mellitus and systemic arterial hypertension, the main causes of CRF, and which should be followed-up in primary care.(15) The fact that black and white patients seek health care only when uremic symptoms start, including abdominal pain, nausea and vomiting, may indicate that they are unaware of the performance of the FHS as a reference for entry into the SUS, and/or that they do not have access to this service. It may also indicate that we have not yet overcome the care model existing prior to SUS, which is based on a merely curative, hospital-centered search and centered on the figure of physicians. However, such aspects do not explain the essence of the findings. After all, we agree with Marx when he says that phenomena and events are simple projections of reality, which was called by Kosik(16) pseudo-concreteness. The search for concrete then goes through the fundamental question: why after thirty years of the construction of SUS, we still have not overcome the hegemonic hospital-centered model of care for people, regardless of their race/color?

It is believed that the explanation of involves the presence of the private sector in the search for diagnosis. Despite being seen as complementary, the interests of the public and private system seem to be antagonistic in many of their nuances, which affect the access to health care for black and white people. This is because in capitalism, health has two contradictory faces: it is both a right and a commodity for generating profit in the private sector. In view of this, we agree with Mészaros(17) when he points out that the capitalist mode of production affects and influences all sectors of society, integrating people, regardless of their race/color, into a larger order.

This higher order seeks an intense accumulation of all socially produced goods, including health, to maintain the power of the ruling class, in the face of the dependence and exploitation of the dominated class. Thus, the presence of the private sector in an important way in the therapeutic itinerary of white and black people with CRF shows that capitalism, transvested in the shape of the private service, was reorganized after the Constitution of 1988 to act in parallel with SUS: while one seeks universality, comprehensiveness and equity, the other acts to generate wealth.(18)

Furthermore, the presence of the private sector demonstrates, intrinsically, how complex the process of alienating people is, since a fetishism has been created in the social imagination, regardless of race/color, which characterizes the private service as efficient, fast and problem-solving, while SUS is represented by queues at hospitals and difficult access. However, the speeches of the participants showed that, although there was the presence of the private sector in the itinerary of white and black people, black people were only diagnosed when the symptoms were evident, which shows that access to the diagnosis was more difficult for this public. In this sense, it is clear that even in the convergences, it is possible to identify marks of institutional racism and inequity.

### Divergences and differences in the therapeutic itinerary of black and white patients and racism materialized in the lack of access for black people

Although racism is present in the convergences, it is believed that it is from the divergences and differences in the therapeutic itinerary that the materialization of institutional racism can be identified in a more expressive way. Racial discrimination in health occurs in a subtle way, little perceived and difficult to be seen. It is implicated in the organizational processes, which result in the difficulty of access, in the negligence that leads to unequal results, something evidenced in the distinct itinerary between the black and non-black groups.(10)

In this category, it is observed that the exclusive access by white people to conservative treatment and to private health plans facilitated access to early diagnosis and better quality of life. Despite the participants, in general, presented CRF as a secondary disease, the self-declared blacks did not undergo routine follow-up in other specialties before the start of hemodialysis, while the self-declared whites did outpatient follow-up with numerous specialties such as cardiology and gynecology, which facilitated the access of this group to the diagnosis of CRF. Another important detail was that despite the symptoms and despite having financial conditions to pay for private consultations (represented by the statement of I7), black people needed hospitalization to be diagnosed with CRF, unlike white people who were diagnosed in outpatient consultations.

Furthermore, white people were the only ones who had access to private health plans, and thus they did not depend exclusively on the public sector. Access to health plans is directly related to people’s financial capacity, whether due to economic power or stability in public employment with the right to health insurance. The public-private offer was discussed by Pilotto and Celeste(19) who found that the double possibility of offer favors people who have a private health plan, and this can generate inequality and inequity when it is taken into account that some people only have the public system as their horizon of access. Therefore, if black people do not have access to private plans, their access to diagnosis is more difficult compared to white people.

According to data published by the National Supplementary Health Agency in August 2019, the sector accounted for 47,332,911 beneficiaries of private health care plans, which corresponded to approximately 22.5% of the population, with greater coverage in the south, southeast and central regions.(20) It is thus noted that having access to private health plans is a privilege achieved by a small portion of the population, which reinforces the perception that it is a privilege that few have access to. Furthermore, according to the Brazilian Institute of Geography and Statistics, in 2016, 75% of the poorest population in the country corresponded to black people.(21) In this perspective, it can be inferred that because the Brazilian black population is mainly in the group of lower purchasing power, it has greater difficulty in accessing private health plans. In this sense, it is possible to see that this population depends exclusively on the State to access socially produced goods, including health care.

According to Marx and Engerls,(22) the State has the primary function of maintaining the status quo, that is, creating rules, laws and organizing society to reduce social tensions, keeping power under the control of a small portion of the population. The State is hardly able to function as an instrument that guarantees rights in its fullness, indirectly naturalizing inequality. Furthermore, it is essential to mention that the current system is based on neoliberalism, which has the precepts of deregulation of the economy, reduction of the State and of social rights and expansion of privatizations, which in practice devaluates the SUS.(23)

It is also worth mentioning that, unlike blacks who started CRF treatment during in-hospital care, a significant portion of white people had access to conservative treatment, in the case of those who were diagnosed early, something fundamental to guarantee the quality of life and prepare to start kidney replacement therapy. Through conservative treatment, people do follow-up with a multidisciplinary team, including specialized physicians, nurses, nutritionists, psychologists and social workers. Thus, it is possible to delay the progression of the disease and improve the outcome in dialysis treatment through the control of comorbidities, prescription of diets and drugs, and choice of the best therapeutic option for the final stage of CRF.(24)

Furthermore, through conservative treatment, patients are psychologically prepared for treatment dependence, in addition to making the vascular access for possible hemodialysis, avoiding more invasive urgent surgical procedures.(24) In this aspect, the lack of access by black people to conservative treatment impacted on their treatment and quality of life, with the onset of early dialysis therapy due to the lack of guidance for controlling the progression of the disease.

From this observation, it is possible to affirm that racism is configured in a system that structures and determines opportunities, negatively impacting on the health conditions of the black people.(25) The problem is not limited to the underdevelopment of Brazil, since the racism has also been discussed as an important factor in the morbidity and mortality of blacks in the United States and the United Kingdom.(26) Thus, it is also noted that racism is a social determinant of health present in several countries with different cultural and economic characteristics.(27)

It is concluded that institutional racism is present in the therapeutic itinerary of people with kidney disease, in which persons of black race/skin color have greater difficulty in accessing health services, negatively impacting the early diagnosis and treatment of CRF. In addition, white people presented a double possibility of assistance through the public and private sector, which allows for better treatment conditions.

However, the transformation of reality goes beyond the verification of the existence of racism. As it is a hegemonic structure, it is believed that the confrontation of institutional racism involves macro- and micro-organizational political articulation. In the macro-organizational scenario, the articulated performance of municipal, state and federal managers is necessary to overcome the hospital-centered model, strengthen primary care and consolidate the PNSIPN. In addition, it is necessary to structure a new economic policy that helps to protect this group and helps them to reach decision-making power in the State.(12) This directly and indirectly requires greater access to income, education and work. In the micro-organizational stance, it is necessary to articulate with social organizations, such as the black movement, with a view to implementing strategies for valuing cultural aspects, expanding the listening channels, identifying and denouncing direct and indirect manifestations of racism.

It is in this micro-organizational context that the present study fits. From the results presented, it was possible to verify that institutional racism negatively impacts the health of the black population. To face this issue, it is necessary to gather efforts of service managers, professionals and users in the search for a change in this paradigm. The study not only provides subsidies to these groups but also contribute to the continuous training of health professionals with a view to transforming daily practices, reducing the neglect of black people and inequities.
